# Traditional Banana Diversity in Oceania: An Endangered Heritage

**DOI:** 10.1371/journal.pone.0151208

**Published:** 2016-03-16

**Authors:** Valérie Kagy, Maurice Wong, Henri Vandenbroucke, Christophe Jenny, Cécile Dubois, Anthony Ollivier, Céline Cardi, Pierre Mournet, Valérie Tuia, Nicolas Roux, Jaroslav Doležel, Xavier Perrier

**Affiliations:** 1 Institut Agronomique néo Calédonien (IAC), Connaissance et Amélioration des Agro-Systèmes, BP 32 98880 La Foa, Nouvelle Calédonie; 2 Service du Développement Rural (SDR), Département de la Recherche Agronomique, BP 100, 98713 Papeete—Tahiti, Polynésie française; 3 Centre de Coopération Internationale en Recherche Agronomique pour le Développement (CIRAD), Unité Mixte de Recherche Amélioration Génétique et Adaptation des Plantes (AGAP), Avenue Agropolis, F-34398 Montpellier Cedex 5, France; 4 Pacific Community, Land Resources Division, Centre for Pacific Crops and Trees (CePaCT), Private Mail Bag, Suva, Fiji Islands; 5 Bioversity International, Parc Scientifique Agropolis II, F-34397 Montpellier Cedex 5, France; 6 Institute of Experimental Botany, Centre of the Region Haná for Biotechnological and Agricultural Research, Šlechtitelů 31, CZ-78371 Olomouc, Czech Republic; United States Department of Agriculture, UNITED STATES

## Abstract

This study aims to understand the genetic diversity of traditional Oceanian starchy bananas in order to propose an efficient conservation strategy for these endangered varieties. SSR and DArT molecular markers are used to characterize a large sample of Pacific accessions, from New Guinea to Tahiti and Hawaii. All Pacific starchy bananas are shown of New Guinea origin, by interspecific hybridization between *Musa acuminata* (AA genome), more precisely its local subspecies *M*. *acuminata* ssp. *banksii*, and *M*. *balbisiana* (BB genome) generating triploid AAB Pacific starchy bananas. These AAB genotypes do not form a subgroup *sensu stricto* and genetic markers differentiate two subgroups across the three morphotypes usually identified: Iholena *versus* Popoulu and Maoli. The Popoulu/Maoli accessions, even if morphologically diverse throughout the Pacific, cluster in the same genetic subgroup. However, the subgroup is not strictly monophyletic and several close, but different genotypes are linked to the dominant genotype. One of the related genotypes is specific to New Caledonia (NC), with morphotypes close to Maoli, but with some primitive characters. It is concluded that the diffusion of Pacific starchy AAB bananas results from a series of introductions of triploids originating in New Guinea area from several sexual recombination events implying different genotypes of *M*. *acuminata* ssp. *banksii*. This scheme of multiple waves from the New Guinea zone is consistent with the archaeological data for peopling of the Pacific. The present geographic distribution suggests that a greater diversity must have existed in the past. Its erosion finds parallels with the erosion of cultural traditions, inexorably declining in most of the Polynesian or Melanesian Islands. Symmetrically, diversity hot spots appear linked to the local persistence of traditions: Maoli in New Caledonian Kanak traditions or Iholena in a few Polynesian islands. These results will contribute to optimizing the conservation strategy for the *ex-situ* Pacific Banana Collection supported collectively by the Pacific countries.

## Introduction

Bananas are widely distributed and cultivated throughout all the Pacific Islands. Various historical traits, through various periods of time and an extended geographical area, led to a diversified range of clonally propagated varieties. All currently cultivated varieties fall into two distinct introduction waves. The most recent wave followed the exploratory travels of Captain Cook at the end of the 18^th^ century, with numerous introductions from almost all the other banana areas in the world [1]. However, it occurred well after a first ‘Oceanian’ wave, which brought traditional starchy bananas to all the Pacific Islands. Like most other cultivated species, these traditional varieties were introduced to the Pacific Islands and their dissemination is directly related to the original eastward peopling of the Pacific from South East (SE) Asia, as described by geneticists, ethnologists, ethnobotanists, linguists and archaeologists [2–8].

The presence of *Homo sapiens* has been attested in the Southeast Asian islands and in near Oceania for at least 50,000 years. Advantage was taken of the very strong drop in sea levels during the different glaciation periods to move from island to island and even easily cross the Wallace line, towards Sahul: New Guinea and Australia (The term ‘New Guinea’ is used here in a generic sense to refer to the country of Papua New Guinea (PNG), the independent state comprising the eastern half of the island of New Guinea and various close islands, as well as the Indonesian western half of the island of New Guinea) [4] At around 30,000 years ago, the islands of the Bismarck Archipelago to the east of New Guinea were already occupied. Around 3,500 years ago, the Lapita cultural complex characterized by its typical “dot-patterned” pottery was developing in the Bismarck Archipelago [5,8]. This Lapita culture, with its language brought by Austronesian mariners, appeared as a complex aggregating innovations borrowed from various SE Asian peoples in the field of stone tools, navigation, but also agriculture [3,7]. Horticulture, in particular, had already been widely developed by the earlier populations, such as at Kuk Swamp, in Highland New Guinea, where tuber plants and bananas were grown 7,000 years ago [9]. Starting from the Bismarck Islands, the Lapita people progressed several thousand kilometres eastwards within a few centuries and settled throughout Melanesia: the Solomon Islands, Vanuatu, New Caledonia, Fiji, and lastly to the western edges of Polynesia, Samoa, where their presence has been attested at 3,000 years ago. Wherever they settled, these migratory proto-Polynesians peoples brought with them their animals [10] and domestic plants [11,12], including many traditional banana cultivars [13,14].

Subsequent, most easterly crossings were much more difficult and it took a further two millennia for the ancestral Polynesians to leave Tonga or Samoa and settle in Tahiti and the Marquesas Islands in 200 AD, then Hawaii around 300–500 AD, Easter Island around 900–1200 AD and New-Zealand only around 1100–1300 AD.

Among the banana varieties specific to the Pacific area, Fe’i bananas, with their distinctive erect bunch, were once important in Tahiti but are today generally under-utilized [15–18]. Fe’i bananas belong to the Autralimusa/Callimusa section (2n = 2x = 20) of genus *Musa* within which a few varieties have been domesticated in the New Guinea area. They are botanically very distinct from other traditional Oceanian bananas, and thus were not considered in this study. Representatives of the Eumusa/Rhodochlamys section (2n = 2x = 22) of genus *Musa* are largely more frequent. They are triploid clones (AAB genome), originating from interspecific hybridization between *Musa balbisiana* (B genome) and *Musa acuminata* (A genome), more precisely *M*. *acuminata* ssp. *banksii*, present mainly in Papua New-Guinea [19,20]. The historical and geographical process of the triploidisation and interspecific hybridization events is fairly well known [21–23].

Three distinct types are usually defined among the Oceanian bananas based on morphological traits: Maoli, Popoulu and Iholena [1,19,24–27]. Popoulu fruits can be easily identified and are short and thick, with a truncate apex; a gradual degeneration of the male bud is often observed as the bunch ripens on the mother plant. Maoli fruits are longer and more slender than Popoulu fruits and are slightly curved, with a blunt tip, and rise up along the bunch axis; the male bud remains generally complete until harvesting, but often weakens. It is also worth noting that intermediate forms exist between these two types, sometimes referred to as Maoli-Popoulu [28]. Iholena fruits are long with a pointed apex, narrowing at both ends compared to the middle of the fruit. The fruits are straight and at right angles to the rachis, giving the bunch a typical appearance. Their colour is light green at flowering but turns to yellow very rapidly two to three weeks before the fruits are fully mature. The lower surface of the leaves (especially the newest unfurling leaf) is typically reddish or silvery and the peduncle may be bright red. The fruit pulp is of a salmon pink coloration. For the three types, fruits are mainly eaten cooked but some of them, particularly for Iholena, which are very sweet when mature, are eaten raw and have greatly appreciated gustatory qualities.

The terminology adopted by botanists for the three morphotypes comes from the names used in the Hawaiian language according to the early typologies proposed for these bananas [25]. ‘Maoli’ with the meaning of “native”, points to the ancient origin of these cultivars, and is equivalent to ‘Maohi’ in Tahiti or ‘Mao’i’ in the Marquesas Islands. Iholena, like its equivalent ‘Ore’a’ in Tahiti or the Marquesas Islands, means “yellow core” and is related to the yellow-orange colour of the fruit pulp. ‘Popoulu’ or ‘Po’u’, ‘Pa’u’ in Tahiti or the Marquesas Islands qualifies the particular shape of the fruit which is often compared to the male flower of the breadfruit [28].

Great diversity is displayed within the Maoli, Popoulu and Iholena types and numerous varieties are recognized and named, within each type on the basis of distinctive secondary morphological characters [25, 28–32]. This diversity of landraces results from a long selection process by farmers who selected varieties for desirable (and in some cases, attractive or spiritually significant) characters. Thus shape, length, fruit and bunch conformations, but also sucker development or length of the production cycle are characters observed by farmers to identify and name a new variety. Some remarkable features, such as leaf variegated colour, pseudostem or petiole colorations are also used. For example, red colorations are often associated with divinities, and some varieties with very red vivid coloration are traditionally reserved for offerings, or for tribal chiefs [33,34].

The Pacific AAB banana plants are sterile triploid clones maintained by vegetative propagation for hundreds, and even thousands of years. Diploid parents of these genotypes are not known, have not been collected until now, or are extinct, or evolved too much to be identified anymore. Moreover, the distinctive characters that motivate the selection and the dissemination of a variety often result from somaclonal mutations and/or epigenetic changes accumulated over vegetative propagation cycles. Within the current scientific knowledge, it is impossible today to reproduce this series of events in a controlled process of varietal improvement. Consequently, it appears essential to develop a conservation management strategy for the current genetic and phenotypic diversity of these bananas. It is an urgent challenge, faced with the current anthropic, environmental and climatic erosion, which is very active on almost all Pacific Islands. At the same time, development issues are multiplying throughout the area. Extension of the varietal panel and dissemination of varieties with original gustative traits or nutritional value [35] are in great demand from part of the population and could gain increased value through the tourism or export sectors with specific fruits such as Popoulu. Moreover the traditional varieties are also often regarded as a cultural heritage which must be preserved for future generations.

The genetic diversity of the traditional Oceanian varieties has been described using several molecular markers [19,20,36–41], but often on samples of small size or not representative of the whole geographical area. Local monographs on these varieties are available [28,30,33,34,42], but no pan-Pacific synthesis has as yet been proposed, thus preventing the construction of a coordinated strategy for an efficient conservation of banana biodiversity. The aim of this study was to contribute to generic scientific knowledge on the genetic diversity of the Maoli, Popoulu, Iholena (MPI) complex, which we technically call Pacific AAB, based on a large sample collected within the various groups of islands in the Pacific and completed with AAB accessions from New Guinea as references. Understanding the diversity of these bananas and the evolution factors acting locally to preserve or reduce that diversity, should make it possible to draw up rational conservation strategies.

## Materials and Methods

No specific permissions were required for these locations/activities as biological samples were sent by CePaCT/SPC and ITC from their *in vitro* collection under the International Treaty on Plant Genetic Resources for Food and Security. This study did not involved endangered or protected species.

**Fig 1:**
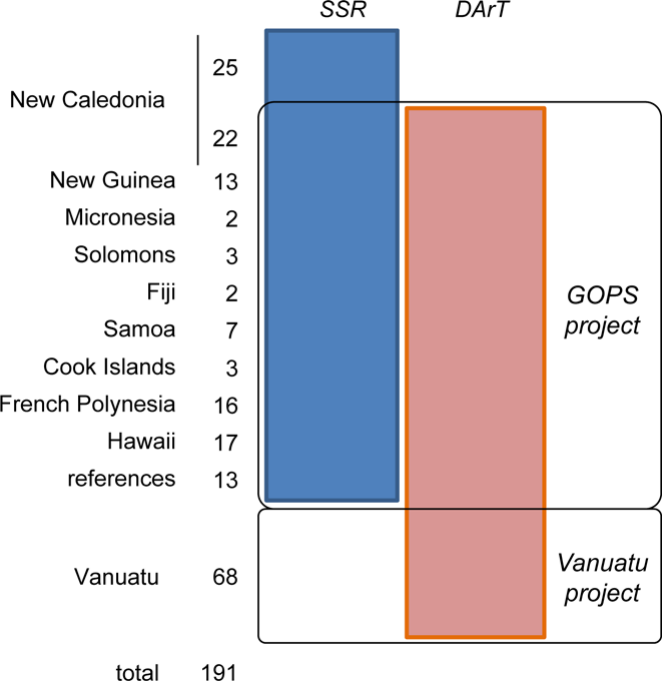
Datasets and supporting research projects.

This study was based on two complementary research projects. The first, devoted to the genetic characterization of diversity within Pacific bananas (GOPS Project, see acknowledgments for acronyms), was complemented with a second project on the genetic variability induced by vegetative propagation on several plant species including bananas in the Vanuatu Islands (Vanuatu Project) as depicted in Fig 1.

### GOPS Project

**Fig 2:**
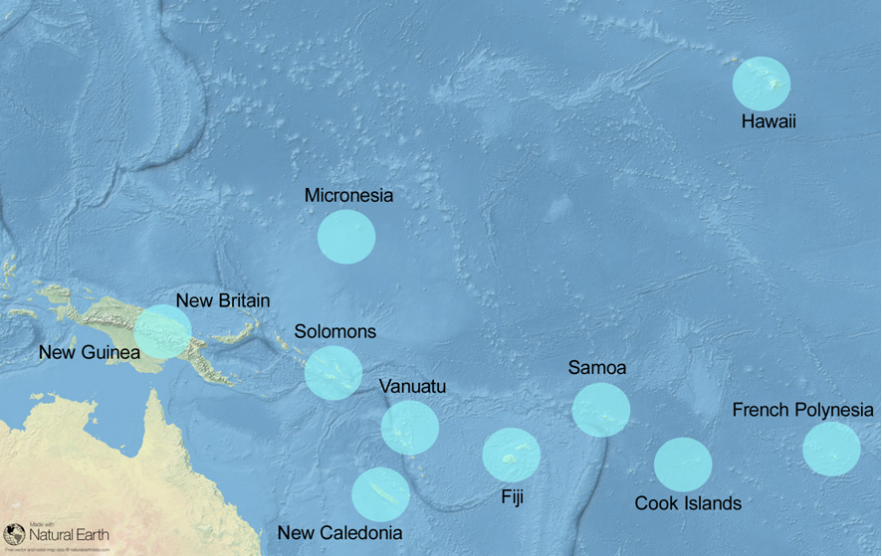
Geographical distribution of the analysed accessions.

A large sample of banana accessions, regarded as belonging to the MPI type, was collected for optimum coverage of the Pacific area (S1 Table for the accession list and Fig 2 for the geographical distribution). Three distant zones were intensively sampled: New Caledonia (22 accessions), French Polynesia (16 accessions), Hawaii (17 accessions). The accessions were sampled from plants maintained *in vivo* and/or in *in vitro* collections, completed in New Caledonia (NC) with collects from ‘farmers’ fields in order to optimize the sampling of the phenotypic diversity observed in various localities within the archipelago. Seventeen accessions were provided by the CePaCT/SPC *in vitro* collection of the Pacific to complete the geographical representativeness of the sample: Cook Islands, Fiji, Samoa, Solomon Islands and Micronesia. Thirteen AAB bananas from New Guinea, were obtained from the International Transit Centre (ITC) in Belgium. Thirteen known representatives of the AA, AAB, ABB subgroups were also added as external references. Later on, in order to confirm the preliminary results, a complementary sample of another 25 presumably MPI bananas was collected in New Caledonia.

### Vanuatu Project

Systematic sampling of bananas cultivated in the Vanuatu Islands was carried out under this project. The hierarchical sampling plan covered six islands of the archipelago, one village per island, 8 farmers par village (selected according to family and age group diversity), with 5 Melanesian gardens per farmer. All varieties declared as grown by the farmer, and not collected from a previous farmer, were sampled. A sample of 68 accessions differentiated by the farmers was gathered; the collection involved all banana types, not just MPI, in order to ascertain the diversity of the bananas currently being grown and to estimate the place of MPI bananas.

### DNA extraction

DNA extraction was performed at IAC New Caledonia, CePaCT/SPC Fiji, SDR French Polynesia and CIRAD Montpellier depending on the availability of the fresh material and sanitary importing rules of each country. The genomic DNA was then isolated and purified either by using the spin column format—DNA Plant Easy Kit (Qiagen, Hilden, Germany), Quick Gene SP-Kit DNA tissue (Fujifilm; Tokyo, Japan) or the CTAB method according to Li et al. (2008) [43]. For the Hawaiian accessions, DNA was isolated from leaf tissues at the Institute of Experimental Botany (Czech Republic). All DNA samples were sent to IAC New Caledonia and CIRAD Montpellier, for Simple Sequence Repeat (SSR) and Diversity Arrays Technology (DArT) genotyping, respectively.

### Genotyping

Two complementary types of molecular markers, SSR and DArT markers, were used to genotype the sampled accessions (Fig 1).

#### SSR genotyping

This was performed at the IAC laboratory in New Caledonia. Twenty-two SSR primer pairs (S2 Table) were selected to analyse the sample grouping the 98 accessions of the GOPS project and the 25 complementary accessions from New Caledonia. Twelve SSRs were developed from *Musa acuminata* ‘*Gobusik’* [44] and the remaining ten were identified from *Musa balbisiana ‘Pisang Klutuk Wulung*’ [45]. These SSRs are known for their good distribution across 10 of the 11 linkage groups [45].

Twenty-five nanograms of *Musa* DNA was PCR amplified in a 96-well Applied Biosystems Thermal Cycler Veriti® with 10 μl final volume of buffer [1X buffer (Promega, USA), 1.5 mM of MgCl_2_, 0.2 mM of dNTPs, 0.1 μM of reverse primer, 0.05μM of forward primer (Eurofins, MWG Operon, Ebersberg, Germany) 0.05 μM of fluorescent labelled forward primer (Applied Biosystems, USA) and 0.4 U of Taq polymerase (GoTaq, Promega USA).

The amplification was performed on a 96-well plate under touchdown PCR conditions: after an initial denaturation at 94°C for 3 min, followed by 10 cycles of 45 s at 94°C, 45 s at annealing temperature, decreasing from 60 to 55°C by 0.5°C each cycle, 72°C for 60 s. Then 24 cycles of 45 s at 94°C, 1 min at 55°C, 45 s at 72°C, followed by a final extension step of 5 min at 72°C.

A size standard covering 35–500 bp (GeneScan^TM^500 LIZ, Applied Biosystems, USA) was added to the PCR products. They were then sized on an AB3130xl Genetic Analyser and genotypes were assigned with GeneMapper ® Software v 4.0 (Applied Biosystems, USA).

After discarding accessions with poor DNA quality, 97 accessions (out of 123) were genotyped with the 22 SSR markers. However, all geographical origins were still present in the genotyped subset, preserving the coverage of the Pacific area.

#### DArT genotyping

Two complexity reduction methods (Pst I/BstNI 16.8%, Pst I/TaqI 16.1%) are available for *Musa* genotypes to generate DArT genomic representations [39]. For these two complexity reduction methods, DArT markers displayed high polymorphism information content. However, when the two methods were calibrated on a panel of 168 *Musa* genotypes, the results showed better quality for Pst I/TaqI representation [39], which was adopted here. The analyses were carried out on the CIRAD genomics platform (Montpellier). DNA samples were genotyped by hybridization on the selected representation array according to Risterucci A.M. *et al* [39].

Polymorphism scoring was carried out using a fluorescent microarray scanner. The score of each marker was calculated using DArTsoft 7.4 (Diversity Arrays Technology P/L, Canberra, Australia), as described by Wenzl et al. (2004) [46]. Markers were scored ‘‘1” for presence, ‘‘0” for absence, and ‘‘X” for indeterminate cases. Several quality parameters were provided for each marker like: the percentage of scored (0 or 1 vs. indeterminate) DNA samples (call rate), the relative between-cluster (‘‘0” vs. ‘‘1”) variance (P value); its multivariate equivalent (Q value) [47] and the reproducibility based on 23 independent repetitions for each marker.

After discarding accessions displaying over-fragmented DNAs, the analysis involved 159 accessions displaying 534 polymorphic markers.

### Data analysis

Data analyses were performed using the DARwin 5.6 software [48].

For SSRs, which are codominant markers, the presence of accessions with different ploidies and the reading ambiguity for triploid loci with 2 alleles (xxy or xyy) made it impossible to use a usual genetic distance measurement for the codominant markers. Here, each marker was dispatched on as many columns as the alleles observed in the population, with a score of 1 or 0 if the allele was present or not. From this disjunctive table, the Dice dissimilarity was calculated between accessions two-by-two on the set of markers present for the two accessions.

For the DArT markers, the presence (1) and the absence (0) of a fragment were given equal weight to calculate the Sokal and Michener dissimilarity index as the proportion of unmatching markers between pairs of accessions. Strict filtering of markers with too much missing data made it possible to ensure a rate of 90% of present data between pairs of accessions.

Diversity trees were built using the weighted Neighbour-Joining (NJ) algorithm [49]. One thousand bootstraps were calculated for the DArT tree. For SSR data, the alleles of the same marker were not independent and random resampling did not make sense.

## Results

### DArT markers

Stringent filtering of the 534 polymorphic markers on the 159 accessions (10 or fewer missing data among the 159 accessions, P> = 80, reproducibility > = 97%) led to retain only 188 markers of very high quality. Ten accessions with more than 20 missing markers out of the 188 were discarded. The final table thus involved 149 accessions characterized by 188 markers.

**Fig 3:**
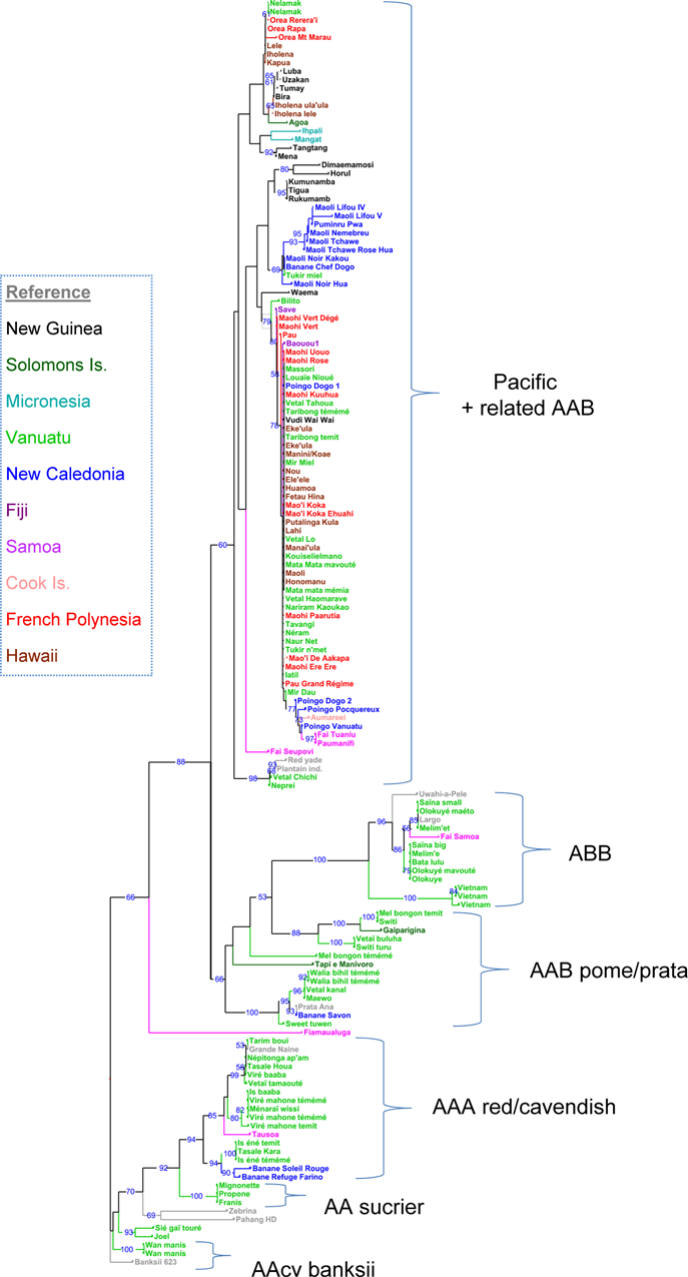
Neighbour-Joining tree on the whole set of 149 accessions genotyped with 188 DArT markers. Rooted on wild diploid acuminata.

Using the dissimilarity matrix calculated from the 149 accessions, an initial diversity tree was constructed on the complete sample (Fig 3). The hierarchical representation was rooted on the cluster including the wild diploids. The references with known genotype were used to interpret the different branches of the tree. The main triploid groups (AAA, AAB, ABB) were represented and among the AAB groups, accessions from the Pacific, associated with the AAB from New Guinea, form a clearly distinct group. Thanks to the exhaustive sampling in the Vanuatu Islands, the analysis showed clearly the place of traditional MPI AAB triploids within the diversity of bananas which are cultivated today.

**Fig 4:**
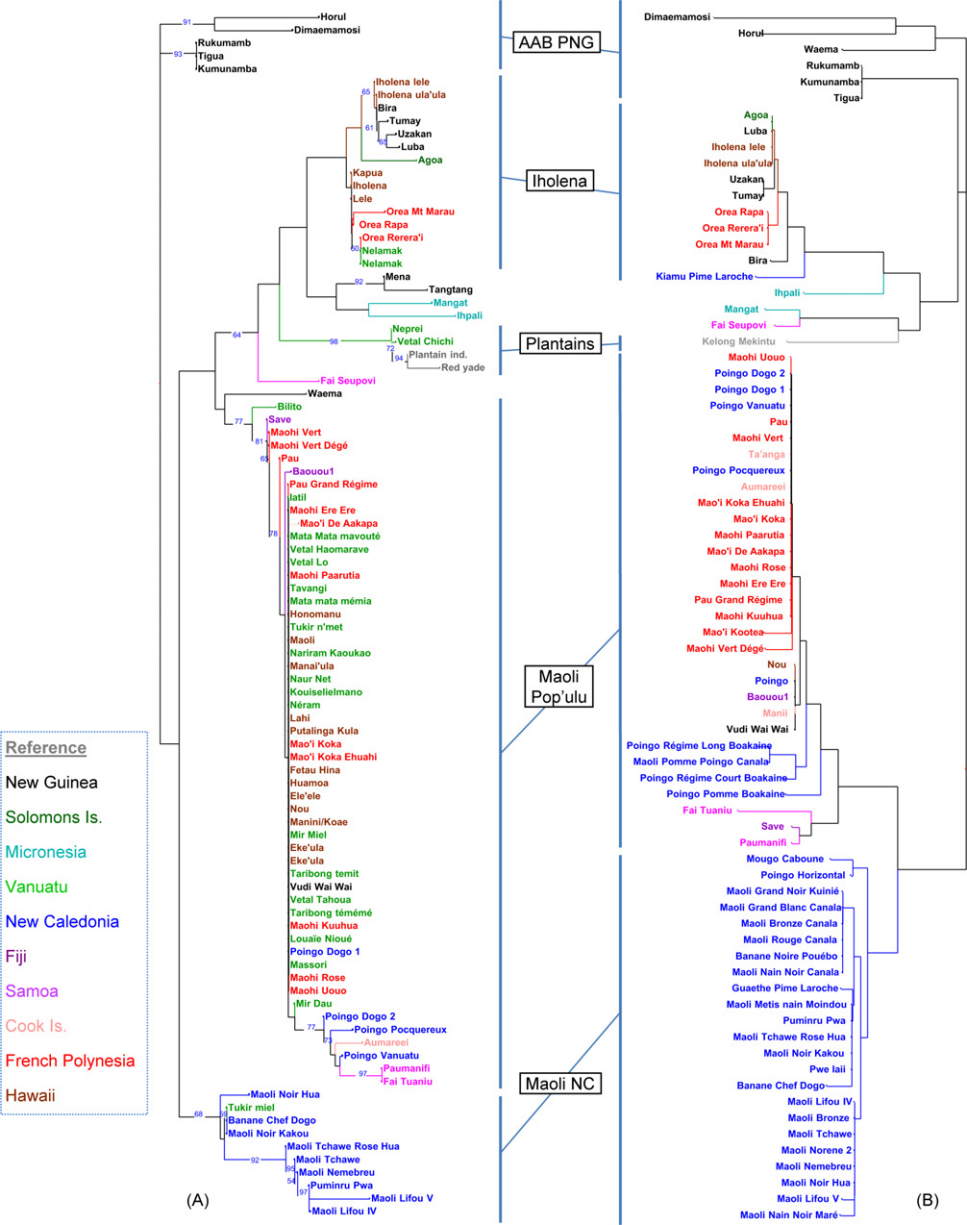
DArT *vs* SSR genetic diversity of Pacific and related AAB bananas. (A) Neighbour-Joining tree from 188 DArT markers on 93 accessions (bootstrap values >50). (B) Neighbour-Joining tree from 21 SSR markers on 75 accessions.

In order to fine-tune this representation, a second tree was restricted to the 93 Pacific accessions and related AAB, including New Guinea AAB and Plantain AAB references (left tree in Fig 4). A very homogeneous set of accessions, from varied geographical origins, represented a majority cluster present throughout the area of distribution. A cluster of genetically more distant forms was specific to New Caledonia. For their part, the Iholena types were also clearly differentiated. Some genetically close, but more original forms, came from specific geographical situations such as Micronesia and Samoa. Lastly, among the New Guinea accessions, several joined the previous clusters but some others formed a genetically more distant cluster.

This structuring within the Pacific AAB is illustrated by the distribution of presences and absences of fragments for each accession (S1 Fig). The markers were ordered according to their increasing values of the total number of presences and locally rearranged manually. The accessions were grouped by profile type. Several blocks of markers appeared where all the markers were identically distributed on the set of accessions. Some of the blocks appeared to be present in the Iholena, or in the Maoli specific to New Caledonia (Maoli NC) cluster, but were absent from the Maoli/Popoulu (MP) cluster, and conversely. These blocks of markers specifically present in, or absent from, Iholena compared to MP were also found in either other Pacific AAB or in the related AAB from New Guinea, or even in the Plantain AAB. Likewise, the blocks that distinguished the Maoli NC from the majority MP group, were found in the accessions from Micronesia, for example, as well as in various AAB from New Guinea, or in the Iholena subset. This structuring in blocks suggests a genetic structuring in several related sets derived from triploidisation events in a genetically differentiated parental pool. It is also worth noting that the DArT markers were not positioned on the *Musa* reference sequences. The blocks of markers could therefore not be assigned to particular chromosomal fragments.

### SSR markers

In order to ensure a rate of at least 80% of present data between pairs of accessions, marker MaCir164 had to be discarded. Using the dissimilarities calculated on 21 markers, a NJ tree (S2 Fig) was constructed on the 97 accessions analysed, including the accessions of the actual GOPS project, and the 25 complementary accessions collected in New Caledonia. This tree was used to select a set of 75 related AAB from the Pacific and New Guinea, discarding the accessions belonging to other genetic groups (ABB, India AAB, etc.).

Although 171 alleles for the 21 microsatellites loci were identified on the whole set of accessions (8.1 alleles/locus, min: 4, max: 14), the allelic richness (A) was only 4.4 alleles per locus when restricted to the Pacific AAB and related accessions. This allelic richness was far less than that observed in [41] on a larger population of *Musa* (14 per locus) with the same marker set. Conversely, on a population of 30 Plantain AAB from Africa, found an allelic richness of 2.4 alleles per locus was found [50]. This narrow genetic base was confirmed using different molecular markers [39,41]. Our results showed limited genetic diversity in Pacific AAB when compared to the whole set of AAB, but higher than in the African Plantain pool, suggesting a broader genetic base.

The NJ tree constructed on the 75 accessions is shown in Fig 4 (right tree). A central cluster of genetically close accessions of varied geographical origins represented the Maoli/Popoulu (MP) type, with several other accessions attached to it, with a few variations maybe due to scoring errors or recent point mutations, which might be frequent for these high mutability markers. Another large, very uniform cluster corresponded to the Maoli NC. The distribution of alleles between the MP and Maoli NC clusters showed that the two sets shared exactly the same alleles for 12 of the 21 markers (S3 Fig). For the other 9, there were always one or two alleles shared between the two groups but with, in addition, one or two specific alleles. However, these specific alleles were always found (to within one exception) in the Iholena and/or in the other Pacific or New Guinea AAB. In comparison, the Iholena and MP clusters shared exactly the same alleles for only 9 of the 21 markers. These results confirm the genetic structuring of these AAB from an ancestral diploid pool, as already shown by the DArT markers.

### SSR and DArT convergence

The two types of markers, SSR and DArT, were used for their complementarity in marking genome diversity. SSR markers are codominant and highly polymorphic. They are very effective in tracing phylogenetic relations between varieties. The limitation is their small number, hence extremely poor coverage of whole genome. DArT are much more numerous, generally several hundred markers that ensure good coverage of the genome. The limitation of these markers is their dominant nature which masks heterozygous situations. This limitation was worsened here as most of the accessions analysed were triploids.

The SSR and DArT analyses involved one common sample of 51 Pacific and related AAB but included, in addition, 24 and 42 specific accessions, respectively. The main structures revealed by the two diversity trees (Fig 4) were nonetheless highly comparable. The conclusions drawn were therefore strengthened, given that the mutational events involved in both cases were different in nature and reflected two types of independent polymorphism.

The main difference came from the relative position of the Maoli NC cluster, which appeared genetically closer to the majority MP cluster by SSR, whereas with the DArT markers, it was only attached after the linking of the MP with the Iholena cluster. We decided to give our preference to the SSR representation which, through the codominant nature of the markers, was more able to approach the phylogenetic relations. The less refined pictures produced from DArT markers have already been highlighted on larger sets of data [39].

## Discussion

The surveys conducted in Vanuatu showed that mostly all the common banana types are grown there today, which is a result that may be extended without any great risk to all the islands of the Pacific. This often involves sweet types, which must have appeared as attractive innovations in this traditional starchy banana zone: the usual dessert AAA of the Cavendish type or similar, the sweet AAB introduced from India, but also the AA of the ‘Sucrier’ type that are found in all production regions. Some starchy bananas of the ABB type are also found which, compared to the traditional AAB, often display greater vigour and an ability to occupy some more marginal zones. This tendency to diversify is also illustrated by the growing of typical African Plantain varieties imported from the Gulf of Guinea. The introduction of diverse cultivars is usually recent and post-colonial; it is historically attested for Cavendish in Hawaii [28], New Caledonia [2,50–54].

The names used in Vanuatu for many of these varieties also indicate recent origins, with qualifiers in the local or Bislama language, a pidgin language with an English lexical base, including some French terms: ‘Vetaï tamaouté’ (= white man’s banana) for the Cavendish AAA, ‘Switi’, ‘Sweet Tuven’ for some sweet AAB, ‘Wan Manis’ (= one month), ‘Franis’ (= French), Mignonette (the French word for sweet AA). In New Caledonia, the bananas introduced since the European era are called ‘Pwi pwagara’ (= white men’s banana) in the Xârâcùù language [34].

These introduced bananas have completed the panel of cultivated varieties, but did not replace traditional MPI bananas which, in Vanuatu, represent the most cultivated subgroup, with around a third of the varieties identified.

More unexpected is the existence in Vanuatu of a few edible AA of the *banksii* type. These starchy diploid bananas, which are very frequent in New Guinea, where they were domesticated more than 7,000 years ago [9], extend very little beyond New Britain and the Solomons [55]. The trace of ancient introductions by migrating populations from the Eastern part of New Guinea can be seen in these few edible AA. They then accompanied the traditional Pacific AAB, but also Fe’i bananas, domesticated from the Australimusa/Callimusa section of genus *Musa* in New Guinea. These AA, which are not very vigorous and have limited adaptation abilities, have not spread very far.

### A New Guinea origin

The concurring results of the analyses for the two types of molecular markers used confirmed that this MPI set is genetically close to African plantains and the AAB from New Guinea which associate a *M*. *acuminata* ssp. *banksii* component and a *M*. *balbisiana* genome. The existence of several New Guinea AAB at the root of the diversity trees supports the origin of these triploids in New Guinea and more precisely, at least for the Maoli and Popoulu, in the Bismarck Archipelago, through hybridization between the local AA *banksii* derivatives and some *M*. *balbisiana* forms brought by human migrations from southern China and the Philippines [56]. The New Guinea AAB can be seen as representatives of bananas taken by the Lapitas in their canoes, no doubt with the starchy edible AA and Fe’i bananas, already mentioned. They then accompanied human migrations as they moved eastwards from the Pacific Ocean [57].

### Iholena versus Maoli / Popoulu

Within our sample, which can be considered geographically representative, the genetic typology clearly isolated the types identified morphologically as Iholena and a few genetically close forms, but did not differentiate between the Maoli and Popoulu types. On this genetic basis, the classification of the MPI in two subgroups is validated Maoli/Popoulu (MP) and Iholena, as considered by other authors [28,30,31,58].

Among the MP varieties, a particular genotypic pattern was largely represented. However, this majority genotype was not morphologically homogeneous, as it included Maoli as well as Popoulu morphotypes. It is present in most of the Pacific Islands (Melanesian or Polynesian), although in New Caledonia it is only represented by some Popoulu types (locally called ‘Poingo’), the others Caledonian MP varieties displaying a quite different genotype.

A few variations around this prevailing genetic pattern brought out some slightly different forms: ‘Fai tuaniu’, ‘Paumanifi’, ‘Save’ from the Central Pacific zone, a few Poingo from New Caledonia, which displayed some original morphotypes. However, these variations were not stable for the two types of markers and are certainly not highly structuring: point mutations or maybe scoring errors for some of them. We chose to consider these variant genotypes as belonging to this majority genetic pattern, perhaps constituting some somaclonal variants.

The distribution of this majority genotype throughout the Pacific can be explained by its propagation accompanying the waves of peopling of the Pacific from PNG. Then, local selections within this genetic pool led to the current phenotypic diversity. Most of the cultivars have large bunches and fingers giving high yield and seem to have been selected for production. To this historical scheme are undoubtedly added many modern exchanges in all directions which have erased the ancient cleavage between Melanesia and Polynesia. Thus, the various Poingo of New Caledonia, which are the only MP bananas to be commercially grown, are fairly typical Popoulu forms and are probably recent introductions.

Phenotypic differentiation between Maoli and Popoulu types is less pronounced in the Western Pacific, where so many intermediate types (Maoli-Popoulu) are found that it was unnecessary for ancient or present islanders to distinguish between Maoli and Popoulu types. This is contrasted with the Eastern Pacific (Hawaii, French Polynesia), where almost all extant cultivars are distinctly Maoli or distinctly Popoulu [28].

However, this majority and ubiquitous genotype was not the only one to be represented within the MP. Several accessions specific to New Caledonia, with also one example from a neighbouring island in southern Vanuatu, were genetically different from the previous group. However, for these two sets, some alleles were shared for all the markers, reflecting a strong genealogical relation. No particular alleles were identified, which would indicate some more exotic hybridizations, and the alleles that differentiated these accessions are present in the Pacific and New Guinea AAB pool. This supports the hypothesis of several original sexual recombination events within the diversified parental AAcv population. These AAB specific to NC are attached to the Maoli morphotype, but often with short bunches, and few and disorderly hands, reminiscent of some more primitive New Guinea forms.

The Iholena type, which was genetically differentiated from the MP, is rare everywhere. Today, Iholena varieties are still present in French Polynesia and in the uplands of Hawaii which, through its human settlement, directly inherited the varietal diversity from the Marquesas and Tahiti. On the opposite side of the geographical spectrum, Iholena types are also found in New Guinea, with some morphologically and genetically identical types. However, today, the Iholena types are extremely rare throughout the intermediate zone. They were found to be genetically close to the Plantain AAB, particularly represented in Africa, but which are thought to originate from the zone between the Philippines and New Guinea (de Langhe, pers. com.). In addition, they are well represented in mainland New Guinea and their presence is attested in islands at west of New Guinea [59,60]. All this leads us to propose a probable origin to the west of New Guinea, or even in the East Indonesian islands. This would then suggest ways of dispersal towards the Pacific that differ from those of the MP.

Some close genetic forms are attested in Micronesia and New Guinea. The more genetically distant accessions ‘Tigua’, ‘Kumunamba’ and ‘Rukumamb’ from New Guinea are original as they are morphologically very similar to the Iholena, with the same typical fruit and bunch shapes, which led to their being classed as Iholena [61], although they do not have the very early yellow coloration of typical Iholena fruits. The similarities of these accessions with the Iholena subgroup are too strong to be down to mere convergence and there is certainly inheritance of a morphological type from a particular subset of parental diploids. This is supported by the observation of these morphological characteristics in various New Guinea AA *banksii*.

### The Pacific AAB do not form a subgroup *stricto sensu*

This Pacific MPI set, sometimes referred to as Pacific Plantains, appears to be genetically variable. It does not therefore constitute a triploid subgroup like the AAB Plantain or the AAA Cavendish subgroups, which are considered to be derived from a single triploidisation event, followed by a long period of diversification through an accumulation of somaclonal variations. The majority genotype of MP cultivars appears to meet this strict definition. However, the MP from NC, the Iholena, the AAB from Micronesia, and the diverse AAB from New Guinea show genetic divergences, even though they arise from the same genetic pool. The diversity linked to the *balbisiana* parent is assumed to be limited [56], so it can therefore be concluded that this observed diversity results from various interspecific triploidisation events in the zone of origin, involving several genetically differentiated AA parents in the vast complex of AA *banksii* diploids. So, unlike the AAB Plantains or AAA Cavendish which diversified through a single event, the Pacific AAB result from a series of introductions of triploids generated in the eastern zone of mainland New Guinea and the New Britain islands for the MP and the western zone of New Guinea (PNG) for the Iholena. This scheme of multiple waves from the New Guinea zone is coherent with the archaeological data for the peopling of the Pacific [17,62].

### Erosion of the diversity

The existence of MP morphotypes with differentiated genotypes mainly in New Caledonia, but also in Samoa, in Micronesia, suggests that greater diversity must have existed in the past. Likewise, the presence of the same Iholena mainly in New Guinea and French Polynesia clearly indicates that these bananas were historically much more abundant and then became less common throughout the Central Pacific zone.

These traditional subsistence bananas, together with other essential Oceanian crop plants such as taro or breadfruit, have travelled thousands of miles with seafarers in their progressive settlement across the Pacific. Banana suckers are able to survive during long maritime crossings; moreover, banana plants have many other uses in addition to the fruit, which were essential in the early settlement time [28,63]. This fundamental initial role is indicated by a constant presence of bananas in the founding myths across the whole Pacific. This long history between humans and banana thus results in its deep embedding in the cultural and ritual background, as attested for example by the richness of associated vocabulary: in Hawaii, more than thirty different terms characterize the various stages of fruit ripening [28]. This long history and the huge geographical dispersion of isolated islands have provided the conditions for wide varietal diversification. The erosion of this diversity began with the introduction from the 19^th^ century of other banana varieties, such as the very popular sweet Cavendish, and the development of a commercial crop in parallel, and in competition, with traditional crops. Associated with that intensification, a suite of pests and diseases (fungi, nematodes, insects, viruses) has decimated many of these traditional productions. Changing lifestyles reducing the demand for traditional crops, and the intensification of land uses (commercial animal and plant production), have hastened this erosion.

Angela Kepler’s work on Hawaiian banana history illustrates this erosion [28]. In Tahiti, twelve different varieties of Iholena were historically attested, while only 4 or 5 still remain. Out of six known Hawaiian Iholena varieties, four are still alive today. Except of the two most common, Lele and Kapua, all are extremely rare, mostly as relics in the upland forest on the island of Maui.

### Diversity hot spots

This erosion of diversity, beginning with the loss of vernacular names, goes hand in hand with the erosion of cultural traditions, which are inexorably declining in the Polynesian Islands (Hawaii, French Polynesia, Samoa, Cook Islands) and to a lesser extent in Vanuatu. In symmetry, diversity hot spots appear to be linked to the local persistence of traditions.

In New Caledonia, Kanak attribute major magical and cultual functions to the different cultivated plants [64]. The “true” banana plants, or “chief bananas”, which are always of the Maoli morphotypes, occupy a prestigious place in the family garden. As they represent the identity of the clan, they must be distinguishable, hence the maintenance of a great diversity of morphological types, including some more primitive morphotypes. They protect important places from “bad” spirits: places of worship, dwellings, but also yam or taro fields. Along with yams and taros, “chief bananas” contribute to customary gifts. Indeed, they all symbolize the wealth of the giving clan and suckers are offered during ritual ceremonies such as weddings. These bananas are grown by the head of family in dedicated plots that are surrounded by many taboos [33]. These Maoli bananas can never been marketed, unlike the Popoulu types (local ‘Poingos’), which do not have any specific status. This alone seems to indicate a more recent introduction, probably during historical movements from Polynesia. The importance of Kanak Custom in New Caledonia might explain the maintenance of primitive Maoli forms in spite of their supposed lower agricultural qualities. Besides, the taboos associated with these banana plants lead to keep them hidden from anyone outside the clan. It is likely that a fair share of the diversity could not be reached during the field surveys.

This status is specific to New Caledonia. The break with Vanuatu is clear, despite the proximity and easy exchanges. Here, tuber crops, yam and taro, play a major role in the social and cultural dynamics and are central to many traditional ceremonies such as weddings, funerals or graduation ceremonies where they are consumed, exhibited and exchanged to establish or reaffirm alliances between communities [53,65]. The role of banana is very limited there today; its presence in wedding gifts is the only indication of an ancient cultural role.

Throughout Polynesia, the magical and ritual function of banana plants has greatly declined. Yet it is clearly mentioned in the notes of James Cook in Tahiti in the 18^th^ Century. It is still perceivable in Hawaii, where it was inherited from the waves of peopling by Marquesan settlers around 100 AD and the first Tahitian settlers after 1100 AD. According to tradition, banana plants were apparently the first plant to have been brought by the founding gods of the Hawaiian peoples [28].

Despite everything, Iholena / Ore’a have retained a special status in a few Polynesian islands. These delicious fruits are still called “bananas of the kings” and are planted around *marae*, sacred sites reserved for social, religious and political activities. Indeed, a local persistence of morphological diversity can be seen, making it possible to distinguish between and name different varieties, within this Iholena group, although today it is rare in the rest of the Pacific. One single example was identified on the island of Mare in New Caledonia. It was the Kiamu accession that the ancient texts describe as *Musa discolor* [33]. This variety plays a very special role since it is strictly reserved for the master of the land to authorize its farming. It also occurs in several legends, with a particular role. It can therefore be imagined that these Iholena morphotypes were sufficiently present, at one time, to be included in the tradition.

### Prospects

With the exception of a few zones where the persistence of traditions has ensured the maintenance of varietal diversity, we found rapid erosion of these traditional crops throughout the Pacific. Faced with such rapid disappearance, we have, based on this work, some foundations for optimizing the conservation of these banana plants that are typical of the Pacific, whether that conservation be *ex situ* in the collections of research organizations, or *in situ* by promoting local conservation. In order to go further in our understanding of factors leading to the maintenance or erosion of diversity, the basic trio of cropping systems in the Pacific needs to be investigated: yam/taro/banana, which have the same geographical origin and have followed the same channels of dissemination, by the same peoples and at the same periods. The breadfruit tree could also be included, as well as sugarcane, which are all vegetatively propagated plants.

These species are culturally associated. In New Caledonia, the “true” bananas are responsible for protecting the “true yams” and the “true taros”. The banana, called the “wife of the yam” is an essential element in traditional yam cultivation: a row of banana plants down the middle of a plot ensures the protection of the yam rows, and indicates the male side of the furrow, which is an important notion in practising the crop. The Kiamu, already mentioned, is associated with yam planting, for which it indicates the important stages of the cropping calendar. As the history of plants is that of the men and women who grow them, an integrated approach of all the plants present in the Melanesian or Polynesian garden becomes self-evident.

The Pacific banana collection is a collective answer of the Pacific countries for the long term conservation strategy of this endangered heritage. This collection hold by the SPC in tissue culture and planted in the field in French Polynesia is the only case of regional full collaborative project for conservation of banana genetic resources. Genetic grouping from SSR and DaRT will be useful to target priority accessions to be added to this collection. This collection is also a support for public education and awareness initiatives, and preservation for next generations.

## Supporting Information

S1 Dataset SSR and DArT Genotyping raw data. (XLSX)

S1 Fig Data representation for 188 DArT markers on 93 Pacific AAB and related AAB. The markers have been ordered in increasing ratio 1/(0+1) on the whole dataset. Alleles 1 are in green, alleles 0 are in blue, missing data are in grey.
(TIFF)

S2 Fig Neighbour-Joining tree on the subset of 97 accessions genotyped with 21 SSR markers. Rooted on diploid acuminata.
(TIF)

S3 Fig SSR allelic distribution of the accessions within MP, Maoli NC, Iholena and other AAB clusters. In blue, alleles shared between MP and Maoli NC. In yellow, alleles present for MP but not for Maoli NC, and, conversely, shared with Iholena or other AAB. In green, alleles specific to MP or to Maoli NC.
(TIFF)

S1 Table List of analysed accessions. (XLSX)

S2 Table List of SSR Markers. (DOC)
