# Supplementary figures and images for: Traditional Banana Diversity in Oceania: An Endangered Heritage
Source: PLoS One. 2016 Mar 16;11(3):e0151208. doi: 10.1371/journal.pone.0151208 (PMC4794170; doi:10.1371/journal.pone.0151208)

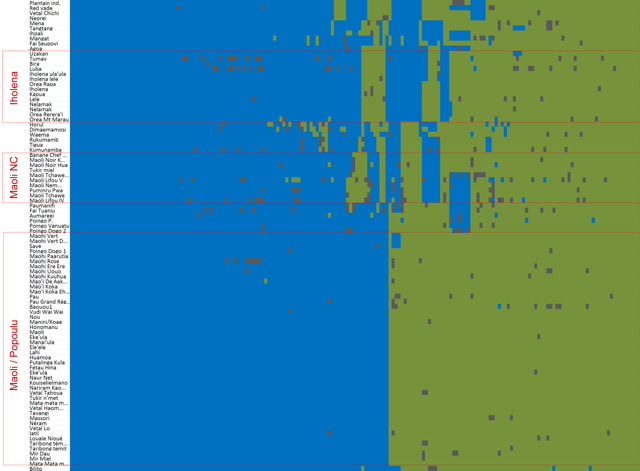

Supplement: S1 Fig — The markers have been ordered in increasing ratio 1/(0+1) on the whole dataset. Alleles 1 are in green, alleles 0 are in blue, missing data are in grey. (TIFF) [file pone.0151208.s002.tiff]

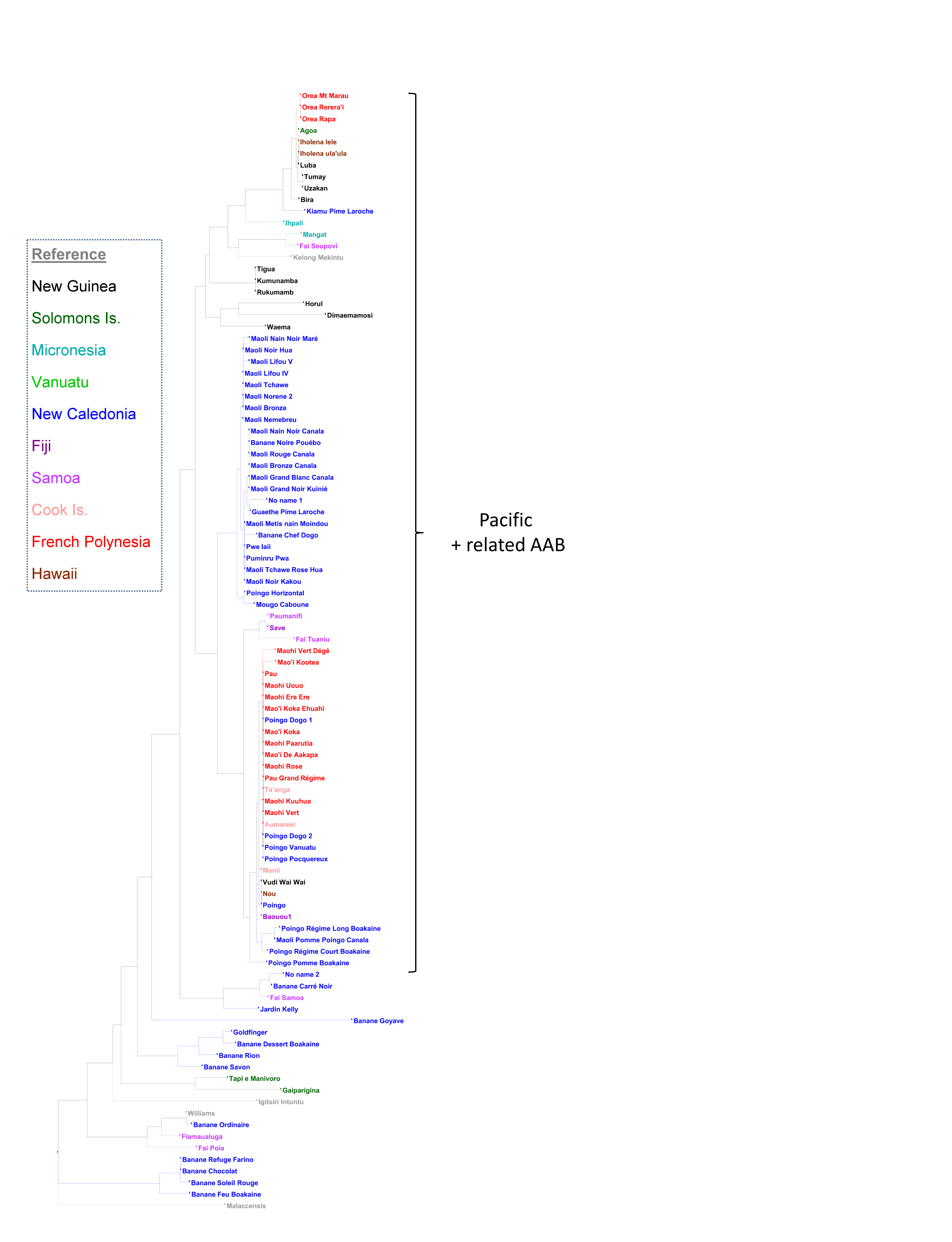

Supplement: S2 Fig — Rooted on diploid acuminata. (TIF) [file pone.0151208.s003.tif]

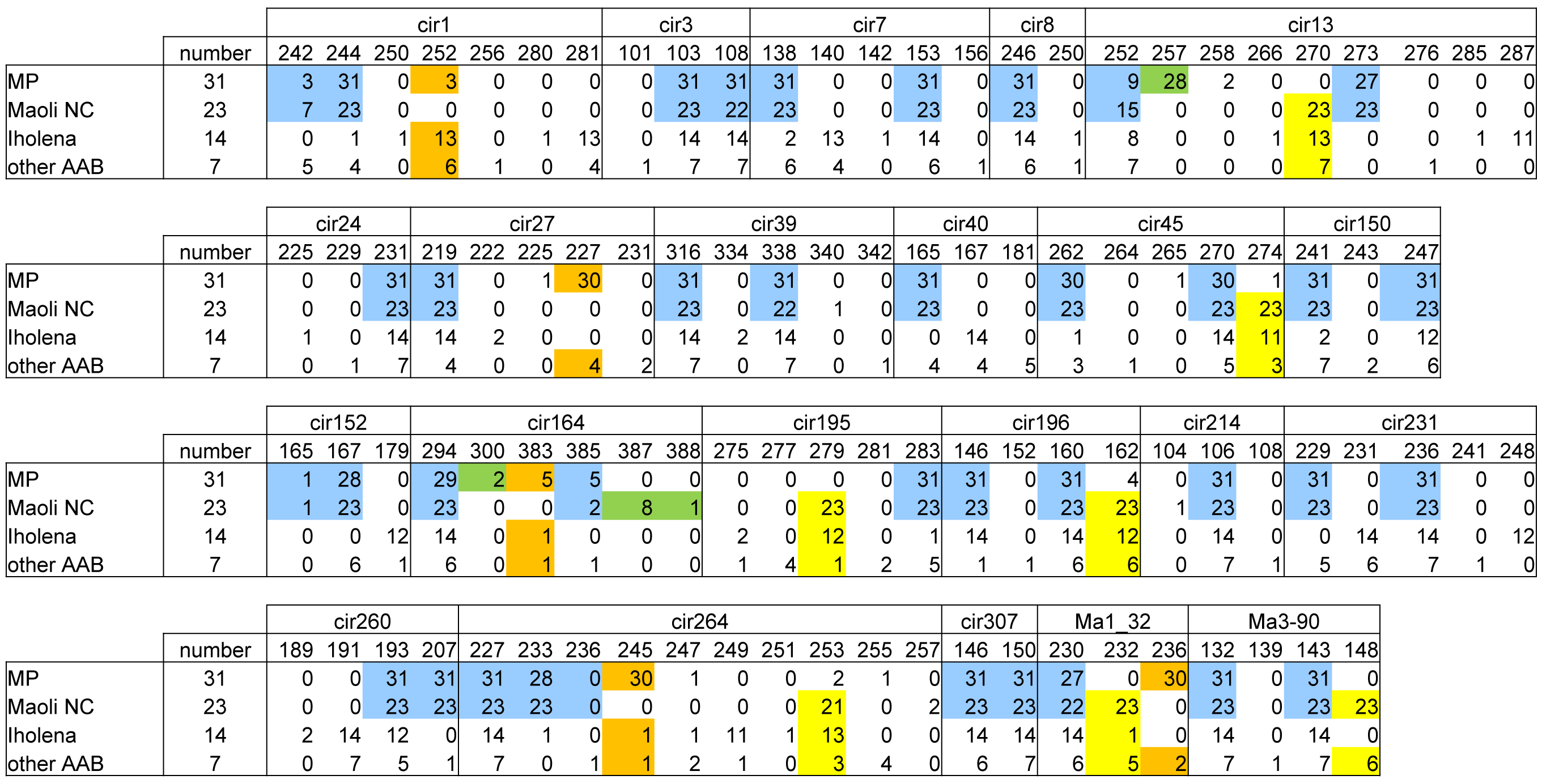

Supplement: S3 Fig — In blue, alleles shared between MP and Maoli NC. In yellow, alleles present for MP but not for Maoli NC, and, conversely, shared with Iholena or other AAB. In green, alleles specific to MP or to Maoli NC. (TIFF) [file pone.0151208.s004.tiff]
