# Supplementary material for: Traditional Banana Diversity in Oceania: An Endangered Heritage
Source: PLoS One. 2016 Mar 16;11(3):e0151208. doi: 10.1371/journal.pone.0151208 (PMC4794170; doi:10.1371/journal.pone.0151208)
Supplement: S2 Table — (DOC) [file pone.0151208.s006.doc]

**S1 Table**. SSR markers

| **SSR** | **Motif** | **min-max size (bp)** |
| --- | --- | --- |
| Ma1-32 | (GA)_17_AA(GA)_8_AA(GA)_2_ | 208–251 |
| Ma3-90 | (CT)_11_ | 123–157 |
| mMaCIR01 | (GA)_20_ | 219–295 |
| mMaCIR03 | (GA)_10_ | 91–119 |
| mMaCIR07 | (GA)_13_ | 127–165 |
| mMaCIR08 | (TC)_6_N_24_(TC)_7_ | 233–279 |
| mMaCIR13 | (GA)_16_N_76_(GA)_8_ | 251–279 |
| mMaCIR24 | (TC)_7_ | 218–278 |
| mMaCIR27 | (GA)_9_ | 212–240 |
| mMaCIR39 | (CA)_5_GATA(GA)_5_ | 310–350 |
| mMaCIR40 | (GA)_13_ | 149–187 |
| mMaCIR45 | (TA)_4_CA(CTCGA)_4_ | 253–275 |
| mMaCIR150 | (CA)_10_ | 238–251 |
| mMaCIR152 | (CTT)_18_ | 139–175 |
| mMaCIR164 | (AC)_14_ | 236–390 |
| mMaCIR195 | (GA)_17_ | 239–295 |
| mMaCIR196 | (TA)_4_(TC)_17_TG(TC)_3_ | 147–173 |
| mMaCIR214 | (AC)_7_ | 96–116 |
| mMaCIR231 | (TC)_10_ | 219–267 |
| mMaCIR260 | (TG)_8_ | 175–211 |
| mMaCIR264 | (CT)_17_ | 215–273 |
| mMaCIR307 | (CA)_6_ | 141–153 |
